# Detecting Genuine and Deliberate Displays of Surprise in Static and Dynamic Faces

**DOI:** 10.3389/fpsyg.2018.01184

**Published:** 2018-07-10

**Authors:** Mircea Zloteanu, Eva G. Krumhuber, Daniel C. Richardson

**Affiliations:** ^1^Department of Computer Science, University College London, London, United Kingdom; ^2^Department of Experimental Psychology, University College London, London, United Kingdom

**Keywords:** facial expressions, posed, emotions, genuineness, accuracy, intensity

## Abstract

People are good at recognizing emotions from facial expressions, but less accurate at determining the authenticity of such expressions. We investigated whether this depends upon the technique that senders use to produce deliberate expressions, and on decoders seeing these in a dynamic or static format. Senders were filmed as they experienced genuine surprise in response to a jack-in-the-box (Genuine). Other senders faked surprise with no preparation (Improvised) or after having first experienced genuine surprise themselves (Rehearsed). Decoders rated the genuineness and intensity of these expressions, and the confidence of their judgment. It was found that both expression type and presentation format impacted decoder perception and accurate discrimination. Genuine surprise achieved the highest ratings of genuineness, intensity, and judgmental confidence (dynamic only), and was fairly accurately discriminated from deliberate surprise expressions. In line with our predictions, Rehearsed expressions were perceived as more genuine (in dynamic presentation), whereas Improvised were seen as more intense (in static presentation). However, both were poorly discriminated as not being genuine. In general, dynamic stimuli improved authenticity discrimination accuracy and perceptual differences between expressions. While decoders could perceive subtle differences between different expressions (especially from dynamic displays), they were not adept at detecting if these were genuine or deliberate. We argue that senders are capable of producing genuine-looking expressions of surprise, enough to fool others as to their veracity.

## Introduction

Facial expressions are an important source of emotional and social information in interpersonal communication. Knowing what another person feels is relevant in predicting someone’s psychological state, likely future behavior, and the outcome of social interactions (Johnston et al., 2010). However, not all expressions are truthful reflections of a person’s underlying emotions. While genuine emotional expressions may inform about the affective state of a person, deliberate or voluntary expressions reflect the strategic intent of the sender in the absence of felt emotions (Ekman and Rosenberg, 2005). For example, deliberate displays can be used to prevent conflict or escalation, spare feelings, reassure, and gain someone’s trust (Ekman and Friesen, 1982). Alternatively, they may be employed to manipulate, deceive, and mask underlying affect or intentions (Ekman and Friesen, 1982). Thus, the ability to discern if someone’s emotional display is genuine or deliberate is of high value in social interaction. The present research explores how different strategies for producing deliberate expressions impact decoders’ perception and ability to detect their authenticity.

Research on emotion recognition has consistently found that decoders are adept at recognizing what emotions are indicated by particular facial expressions (Ekman, 2003; Calvo and Nummenmaa, 2015). But, when it comes to judging the authenticity of such facial displays, accuracy rates are markedly lower (Frank and Ekman, 1997; McLellan et al., 2010). When judging deception, for example, they are often at chance levels (Bond and DePaulo, 2006; Porter et al., 2012). This raises questions regarding the role emotions play in communication and social interactions. People regularly produce expressions when they wish to communicate to another person how they feel (Zuckerman et al., 1986). However, the advantage of decoding such expressions hinges on the displays matching the senders’ true underlying affect. For instance, liars in real-world high-stakes scenarios have been shown to produce deliberate expressions to aid their deception, which decoders are unable to differentiate from genuine expressions (Porter et al., 2012). This is compounded by the fundamental assumption decoders make that the behavior of others is honest, unless prompted to consider otherwise (DePaulo and DePaulo, 1989). If decoders cannot distinguish deliberate displays from genuine affect these may be used to the advantage of the sender (i.e., lying about one’s feelings), leading to misleading or even detrimental inferences. The case may be that senders are capable of producing deliberate expressions that resemble genuine affect sufficiently to fool decoders (Krumhuber and Manstead, 2009). Thus, it is important to understand if human decoders can discriminate genuine and deliberate expressions of emotions.

In the past, much of the emotion perception work attempting to answer this question has focused on a binary distinction between spontaneous (genuine) and posed (deliberate) expressions. To this end, a variety of acted expressions have been considered under the umbrella term of ‘posed’ displays, thereby glossing over different production methods that may lead to differences in expression and perception. Such voluntary behavior has typically been thought to differ from spontaneous expressions in the neural pathways of cortical and subcortical activation (Rinn, 1984; Morecraft et al., 2001), resulting in marked differences in visual appearance and timing (Cohn and Schmidt, 2004; Namba et al., 2016).

Whilst existing research suggests deliberate displays offer an advantage in emotion recognition tasks (Dawel et al., 2016), their use has been criticized in recent years due to their intentional nature to communicate the desired emotion (see Sauter and Fischer, 2017). Given the prevalence of existing stimulus sets to feature voluntary facial expressions (for a review see Krumhuber et al., 2017), we think it is important to draw a difference between various types of deliberate behavior. For example, the classical ‘posed expressions’ are voluntarily-produced emotional displays resulting from specific instructions such as those employed in directed facial action tasks (Russell, 1994). ‘Portrayed expressions’ are spontaneous deliberate expressions that occur in the absence of explicit instructions, but are congruent with the context in which they occur, such as smiling for a photograph (Vazire et al., 2009). ‘Enacted expressions’ are expressions voluntarily produced after reliving a congruent past experience of the emotion, often done using method acting techniques (Scherer and Bänziger, 2010). Furthermore, the way in which researchers produce emotional displays for their stimuli vary widely, from using photographic stimuli that senders must imitate (e.g., Field and Walden, 1982), to the direct manipulation of facial muscle activation (e.g., Ekman et al., 1983), or simply using verbal prompts (e.g., Lewis et al., 1987). Thus, a further goal of our research is to shed light on the effect that these different practices may have on how human emotion perception is studied.

Accounting for this large variability in production methods, it seems reasonable to explore the impact of these different types of deliberate displays on expression perception. For this, we focused on the perception of a single emotion: surprise. Surprise is considered a basic emotion, having a distinctive facial configuration that is well recognized cross-culturally (Nelson and Russell, 2013; Namba et al., 2016). It is consistently found to have high recognition rates, second only to happiness (Ekman, 2003). Also, surprise is argued to be a neutral-valence emotion, and one determined by context (Ekman, 2004). In order to elicit surprise spontaneously, we considered the surprise expression to be more closely related to the startle response, i.e., a sudden defensive response to an external aversive stimulus. We therefore used a jack-in-the-box, an approach that in the past has been successful in eliciting a startle response, primarily in infants (e.g., Reissland et al., 2002), due in part to the unpredictable timing and the abrupt appearance of the jack. In addition to genuine expressions of surprise, two types of deliberate expressions were produced either on the basis of a recent emotional experience, or via improvisation based on no/minimal information.

Besides considering expression type, we investigated whether the modality of presentation (static vs. dynamic) can significantly impact authenticity discrimination. While static facial expressions of adequate intensity are sufficient to allow accurate emotion classification, dynamic aspects have been shown to enhance ratings of naturalness (Sato and Yoshikawa, 2004) and intensity (Biele and Grabowska, 2006), leading to stronger facial mimicry (Sato et al., 2008) and brain activation patterns in decoders (Trautmann et al., 2009). Dynamic information also enables better discrimination between genuine and deliberate displays (Krumhuber and Manstead, 2009; Maringer et al., 2011). This may be due to the fact that these are more complex and richer in expressive signal, thereby helping with the processing of emotional information (see Krumhuber et al., 2013). The use of dynamic stimuli may consequently better reflect the true authenticity of an expression.

In the present research, we contrasted genuine expressions of surprise with deliberate expressions produced after seeing an affect-evoking stimulus, i.e., the jack-in-the-box (Rehearsed) or without seeing it (Improvised). Re-enacting a genuine emotional experience is thought to facilitate the production of an authentic-looking deceptive display, as the sender is using the recent affective information of how an emotion feels and makes them behave (Bänziger and Scherer, 2007). This in turn may produce an expression that closely mirrors spontaneous surprise. Alternatively, improvising an expression by using one’s lay beliefs may produce a successful deceptive display (cf. Reisenzein et al., 2006), as the aim is to convey a specific message, which in turn may match the expectations of the decoder (i.e., exaggerated expressions are better recognized; Hess et al., 1997).

We hypothesized differences between the three types of surprise expressions in terms of their perceived genuineness, intensity, and judgmental confidence. Specifically, decoders should be able to accurately and confidently detect genuine surprise (Genuine), but should show poorer performance and less confidence when judging deliberate expressions (Rehearsed and Improvised). Whilst rehearsed surprise might lead to higher ratings of genuineness in comparison to improvised surprise, it is the improvised expressions that are predicted to be perceived as higher in intensity.

These differences in expression perception should be further moderated by the presentation format (static vs. dynamic). Using dynamic stimuli compared to static images stimuli increases ecological validity, allows for subtler elements of an emotion (e.g., onset, timing, duration, and fluidity) to be incorporated into the decoding process, and can improve authenticity discrimination (e.g., Hess and Kleck, 1994; Ambadar et al., 2005; Krumhuber and Kappas, 2005). We therefore predicted that dynamic information enables a better discrimination between genuine and deliberate expressions than what could be achieved with static displays.

## Materials and Methods

### Participants

A total of 120 participants were recruited online through Amazon’s Mechanical Turk (MTurk^1^) in exchange for $0.75; MTurk was used due to the benefits offered by online recruitment, and the comparable responses to laboratory samples (see, Casler et al., 2013). After deleting incomplete cases (*N* = 31) the final data encompassed 89 participants (51 men, 38 women), with an age range of 20–54 years (*M* = 29.9, *SD* = 8.9). Informed consent was obtained online prior to their participation. The two-factor experimental design included the presentation format (static vs. dynamic) as between-subjects variable, and expression type (genuine, rehearsed, and improvised) as within-subjects variable. Participants were randomly assigned to one of the two conditions, resulting in 46 people in the static group and 43 people in the dynamic group. A power analysis using G^∗^Power 3.1 (Faul et al., 2007) for an interaction between presentation format (2) and expression type (3), assuming a medium-sized effect (Cohen’s *f* = 0.18), determined that this sample size would be sufficient for 95% power. All participants had normal or corrected-to-normal vision. Ethical approval for the present study was granted by the UCL Department of Psychology Ethics Committee.

### Stimulus Material

For the production of the stimulus expressions of surprise, 39 university students (12 males, 27 females; *M*_age_ = 24.54, *SD* = 5.31; age range = 19–45 years) were video-recorded under one of the three elicitation conditions:

In the *Genuine* condition, encoders were seated in front of the jack-in-the-box and turned the wheel until the toy “popped out”; a melody played as the wheel was turned prompting the action from the toy. The exact function of the toy was not described prior to the start of the experiment nor was the emotion under investigation explicitly mentioned. A camera was placed at eye-level, and recoded their reaction from the start of the winding action until the end of their behavioral response; the jack was not visible in the videos.

In the *Improvised* condition, encoders turned the wheel, carrying out the same hand action as those in the genuine videos. However, the electronic mechanism that releases the toy was made non-operational. Instead participants watched a video on a tablet positioned in front of the box. The video showed a countdown and played the same melody as the jack-in-the-box. When the word “NOW” appeared on the screen, participants had to act in a surprised manner. The countdown was matched for time and volume with the jack-in-the-box.

In the *Rehearsed* condition, encoders first had the experience of seeing the real jack-in-the-box as those in the genuine condition. The jack’s wheel was then disconnected from the releasing mechanism, and the tablet with the countdown video was placed in front of it, as done in the Improvised condition. This time, encoders were asked to reproduce their previous emotional reaction when the word “NOW” appeared on the tablet’s screen.

**FIGURE 1:**
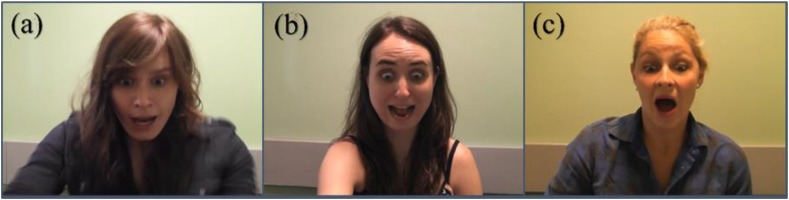
Stimuli used in the study illustrating the three types of surprise expressions: **(a)** Genuine, **(b)** Rehearsed, and **(c)** Improvised.

A Panasonic SDR-T50 camcorder was used to record the facial reactions at 25 frames per second. For each condition, there were thirteen exemplars: Genuine (4 men, 9 women), Rehearsed (5 men, 8 women), and Improvised (3 men, 10 women). These produced both static and dynamic portrayals of each expression, netting 39 static and 39 dynamic stimuli. Dynamic stimuli were silent video clips and lasted approximately 10 s. Static stimuli consisted of a single frame of the peak expression taken from each video; defined as the frame before the expression began to relax (see **Figure 1**). All stimuli were displayed in color (size: 1920 pixels × 1080 pixels).

### Procedure

The study was conducted using the Qualtrics software (Provo, UT). As mood can affect classification accuracy (Forgas and East, 2008), it was necessary to control for this factor, by asking participants the following question: “How do you feel at this moment?” using a 5-point Likert-type scale (1 – *extremely sad*, 5 – *extremely happy*). After obtaining age and gender information, they were instructed to watch each stimulus carefully and rate the facial expression of the sender. It was made clear that some senders were genuinely reacting to a jack-in-the-box, while others never saw the toy puppet popping out and were merely attempting to appear surprised. Participants saw either static or dynamic displays of all 39 stimuli (presentation duration was 10 s in both conditions), in randomized order, and rated the expressions on several dimensions.

The extent to which they perceived the expression as a *genuine* response to seeing the jack-in-the-box was measured using a single item, 5-point Likert-type scale ranging from -2 (‘*certain no Jack-in-the-box*’), -1 (‘*no Jack-in-the-box’*), midpoint of 0 (‘*not sure*’), to +1 (‘*with Jack-in-the-box*’) and +2 (‘*certain with Jack-in-the-box*’), with higher scores indicating greater perceived genuineness. The responses were aggregated across the 13 exemplars of an expression type, yielding a total score ranging from -26 to +26 on perceived genuineness (see Dawel et al., 2016).

Overall *accuracy* of participants’ ratings of the expressions were also calculated. A judgment was accurate if participants responded that they thought there was a jack-in-the-box present (with any level of certainty) and indeed the sender was reacting to a jack-in-the-box, or if they responded that there was no jack-in-the-box and, in fact, the sender was only pretending to be surprised. To formulate the measure of accuracy in authenticity discrimination, these responses were compared to the actual conditions of the stimulus, ignoring trials in which the participant responded ‘not sure’ (see Levine et al., 1999). If there was a match (e.g., rehearsed and improvised expressions were seen as having no jack-in-the-box, and genuine expressions were judged to have a jack-in-the-box), they were coded as accurate (score = 1). If there was a mismatch, it was coded as inaccurate (score = 0), yielding a final total score ranging from 0 to 13 for each expression type. For ease of comprehension, we re-labeled the totals using a percentage scale from 0% (lowest accuracy) to 100% (highest accuracy).

This was followed by participants’ *confidence* ratings of their decision (1 – *not at all*, 5 – *very much*) to assess potential discrepancies between accuracy and perceived ability (Vrij and Mann, 2001). Finally, participants were asked to judge the *intensity* of the sender’s expression using a 5-point Likert-type scale (1 – *not at all*, 5 – *very much*).

## Results

Preliminary analyses indicated no significant differences between male and female participants in their judgment ratings, *F*s < 1.95, *p*s > 0.15. Thus, we collapsed across gender for all subsequent analyses. Adding mood as a covariate did not affect any of the results reported below, *p*s > 0.30. In both conditions, judgment ratings were averaged across the 13 exemplars within each expression type. A 2 (Format: dynamic vs. static) × 3 (Expression: genuine, improvised, rehearsed) mixed-factorial ANOVA was conducted on each of the four dependent measures. The Greenhouse–Geisser adjustment to the degrees of freedom was applied when Mauchly’s test indicated that the assumption of sphericity had been violated.

### Genuineness

**FIGURE 2:**
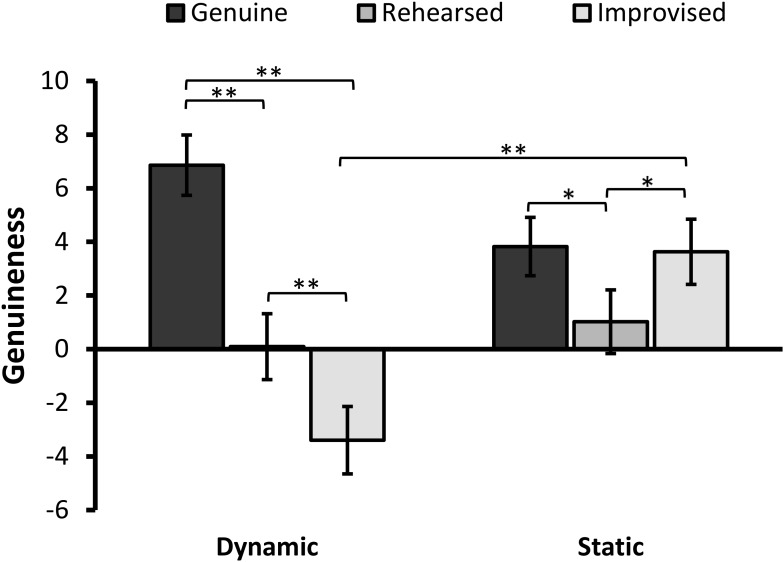
Mean ratings for perceived genuineness of facial expressions (error bars ±1 SE). Positive values indicate that expressions were perceived as more genuine, while negative values indicate that they were perceived as more fake. The asterisks represent a significant difference at ^∗^*p* < 0.005 and ^∗∗^*p* < 0.001.

There was a significant main effect of Expression, *F*(1.81,157.72) = 39.78, *p* < 0.001, $\eta_{p}^{2}$ = 0.314, but not Format, *F*(1,87) = 1.20, *p* = 0.277, on perceived genuineness. In addition, the interaction between the two factors was significant, *F*(1.81,157.72) = 30.40, *p* < 0.001, $\eta_{p}^{2}$ = 0.259 (see **Figure 2**). To decompose the interaction, the simple main effect of expression was analyzed on each format condition.

The results revealed a significant simple main effect of Expression in the dynamic condition, *F*(2,86) = 49.45, *p* < 0.001, $\eta_{p}^{2}$ = 0.535. Pairwise comparisons with Bonferroni correction showed that genuine expressions (*M* = 6.86; *SD* = 5.49) were rated as significantly more genuine than improvised expressions (*M* = -3.40; *SD* = 7.60), *t*(42) = 9.68, *p* < 0.001, 95% CI [8.12, 12.39], *d* = 1.48, and rehearsed expressions (*M* = 0.09; *SD* = 7.40), *t*(42) = 6.69, *p* < 0.001, 95% CI [4.73, 8.81], *d* = 1.02. Improvised expressions were judged to be the least genuine and significantly differed from rehearsed expressions *t*(42) = -5.23, *p* < 0.001, 95% CI [2.14, 4.84], *d* = 0.80.

The analysis also revealed a significant main effect of Expression in the static condition, *F*(2,86) = 7.76, *p* = 0.001, $\eta_{p}^{2}$ = 0.153. Pairwise comparisons revealed that genuine expressions (*M* = 3.83; *SD* = 8.78) were rated significantly more genuine than rehearsed expressions (*M* = 1.02; *SD* = 8.61), *t*(45) = 3.02, *p* = 0.004, 95% CI [0.93, 4.67], *d* = 0.45, but no different from improvised expressions (*M* = 3.63; *SD* = 8.80), *t* < 1, *p* = 0.839. Improvised expressions were also judged as significantly more genuine-looking that rehearsed expressions, *t*(45) = 3.14, *p* = 0.003, 95% CI [4.28, 0.94], *d* = 0.47.

When considering differences in genuineness ratings between formats, simple effects analyses showed that improvised expressions were judged as significantly less genuine-looking when they were presented in dynamic than static format, *F*(1,87) = 16.14, *p* < 0.001, $\eta_{p}^{2}$ = 0.156. This difference did not occur in the context of genuine, *F*(1,87) = 3.76, *p* = 0.056, $\eta_{p}^{2}$ = 0.041 or rehearsed expressions, *F* < 1, *p* > 0.59.

### Accuracy

**FIGURE 3:**
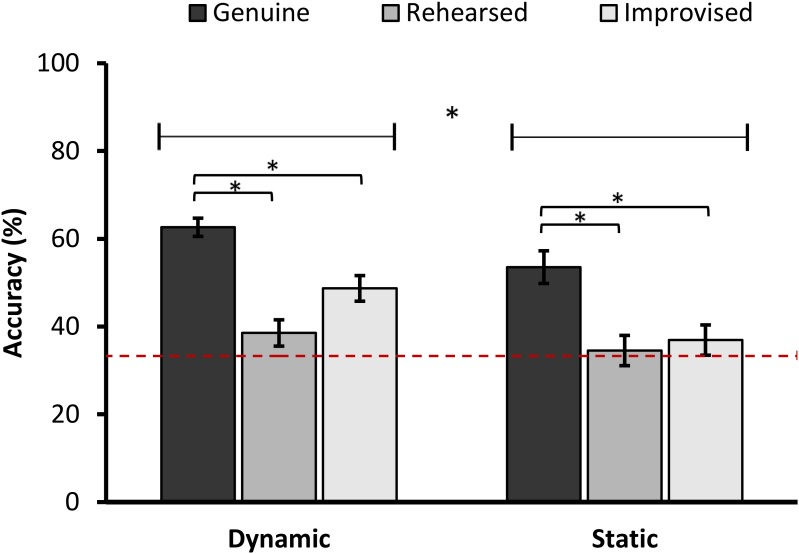
Mean accuracies in authenticity discrimination in the dynamic and static format split by expression type (error bars ±1 SE). The lines above the bars represents a main effect of Format. The brackets above the bars represent a significant difference between Expression type. The asterisks represent a significant difference at *p* < 0.001. The dotted line represents chance performance (33.3%).

The ANOVA showed a significant main effect of Expression, *F*(1.23,106.94) = 22.08, *p* < 0.001, $\eta_{p}^{2}$ = 0.202, and Format, *F*(1,87) = 10.70, *p* = 0.002, $\eta_{p}^{2}$ = 0.109. Overall, accuracies in authenticity discrimination were higher in the dynamic than static condition (*M*_diff_ = 8.34, *SD*_diff_ = 2.55). Also, genuine expressions (*M* = 57.92, *SD* = 20.85) were rated more accurately than both rehearsed (*M* = 37.92, *SD* = 21.69), *t*(88) = 5.23, *p* < 0.001, 95% CI [1.16, 3.11], *d* = 0.55, and improvised expressions (*M* = 41.46, *SD* = 22.46), *t*(88) = 4.37, *p* < 0.001, 95% CI [1.64, 3.11], *d* = 0.46. The difference in accuracy between rehearsed and improvised expressions was not significant, *t*(88) = 2.23, *p* = 0.028, 95% CI [0.05, 0.87], *d* = 0.24. The interaction term was not significant, *F*(1.23,106.94) = 1.72, *p* = 0.193 (**Figure 3**). When comparing the accuracy scores to chance performance (33.3%), genuine expressions were classified significantly above chance level, *t*(88) = 11.14, *p* < 0.001, 95% CI [2.63, 3.77], *d* = 1.18, as were improvised expressions, *t*(88) = 3.42, *p* = 0.001, 95% CI [0.44, 1.68], *d* = 0.36. However, rehearsed expressions were no different from chance (Bonferroni corrected), *t*(88) = 2.01, *p* = 0.048, 95% CI [0.01, 1.19].

### Intensity

**FIGURE 4:**
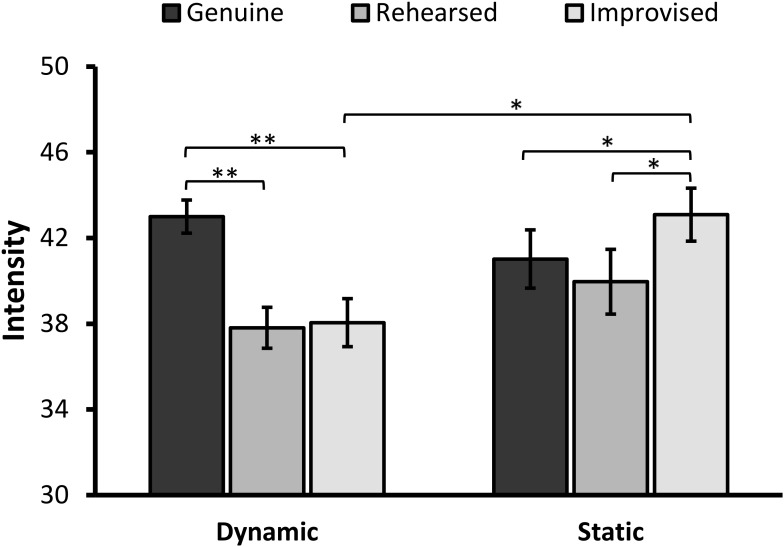
Mean ratings for perceived intensity of facial expressions (error bars ±1 SE). The asterisks represent a significant difference at ^∗^*p* < 0.01 and ^∗∗^*p* < 0.001.

There was a main effect of Expression, *F*(2,174) = 15.72, *p* < 0.001, $\eta_{p}^{2}$ = 0.153, but no effect of Format, *F*(1,87) = 1.22, *p* = 272, on perceived intensity. The interaction between the two factors was significant, *F*(2,174) = 19.98, *p* < 0.001, $\eta_{p}^{2}$ = 0.187 (**Figure 4**).

When decomposing the interaction, simple effects analyses revealed a significant main effect of Expression in the dynamic condition, *F*(2,86) = 25.38 *p* < 0.001, $\eta_{p}^{2}$ = 0.371. Pairwise comparisons with Bonferroni correction showed that genuine expressions (*M* = 43.00, *SD* = 5.07) were rated as more intense than rehearsed (*M* = 37.81, *SD* = 6.27), *t*(42) = 6.63, *p* < 0.001, 95% CI [3.61, 6.77], *d* = 0.70, and improvised expressions (*M* = 38.05, *SD* = 7.34), *t*(42) = 5.57, *p* < 0.001, 95% CI [3.16, 6.75], *d* = 0.59. Both types of deliberate expressions did not, however, significantly differ from each other, *t* < 1, *p* > 0.99.

Additionally, a significant simple main effect of Expression in the static condition, *F*(2,86) = 8.59, *p* < 0.001, $\eta_{p}^{2}$ = 0.166, showed that genuine expressions (*M* = 41.02, *SD* = 9.24) were rated as less intense than improvised expressions (*M* = 43.09, *SD* = 8.39), *t*(45) = -2.84, *p* = 0.007, 95% CI [-3.53, -0.60], *d* = 0.30, but not rehearsed expressions, *t*(45) = 1.35, *p* = 0.183, 95% CI [-0.53, 2.65]. Improvised expressions were perceived as more intense than rehearsed expressions, *t*(45) = 3.21, *p* = 0.002, 95% CI [1.17, 5.10], *d* = 0.34.

When considering differences in intensity ratings between formats, simple effects analyses showed that improvised expressions were judged as significantly more intense when they were presented in dynamic than static format, *F*(1,87) = 9.05, *p* = 0.003, $\eta_{p}^{2}$ = 0.094. This difference did not occur in the context of genuine, *F*(1,87) = 1.54, *p* = 0.218, $\eta_{p}^{2}$ = 0.017, or rehearsed expressions, *F*(1,87) = 1.39, *p* = 0.241, $\eta_{p}^{2}$ = 0.016.

### Confidence

**FIGURE 5:**
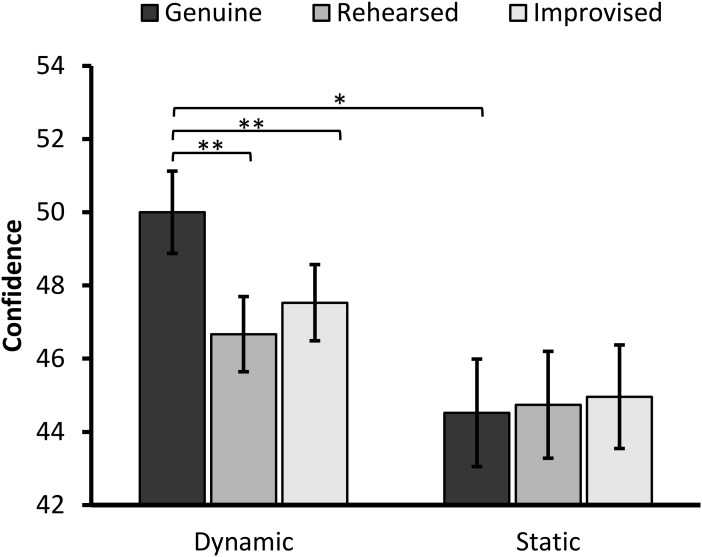
Confidence scores in the dynamic and static format split by expression type (error bars ±1 SE). The asterisks represent a significant difference at ^∗^*p* < 0.01 and ^∗∗^*p* ≤ 0.001.

The ANOVA revealed a main effect of Expression, *F*(2,174) = 6.14, *p* = 0.003, $\eta_{p}^{2}$ = 0.066, and a marginal significant effect of Format, *F*(1,87) = 3.66, *p* = 0.059, $\eta_{p}^{2}$ = 0.040, on confidence ratings. These effects were qualified by a significant interaction between the two factors, *F*(2,174) = 8.78, *p* < 0.001, $\eta_{p}^{2}$ = 0.092 (**Figure 5**).

When decomposing the interaction, the simple main effect of Expressions was significant in the dynamic condition, *F*(2,86) = 14.29, *p* < 0.001, $\eta_{p}^{2}$ = 0.249. Pairwise comparisons with Bonferroni correction showed that participants were less confident in their ratings of rehearsed (*M* = 46.67, *SD* = 6.74) and improvised expressions (*M* = 47.53, *SD* = 6.83), compared to genuine expressions (*M* = 50.00, *SD* = 7.48), *t*(42) = 4.13, *p* < 0.001, 95% CI [1.70, 4.95], *d* = 0.44, *t*(42) = 3.76, *p* = 0.001, 95% CI [1.14, 3.79], *d* = 0.40. The two deliberate expressions did not significantly differ from each other, *t*(42) = 1.11, *p* = 0.27, 95% CI [-0.70, 2.42].

The simple main effect of Expression was not significant in the static condition, *F* < 1, *p* > 0.75.

When considering differences in confidence ratings between formats, simple effects analyses showed that genuine expressions were more confidently judged in the dynamic than static condition, *F*(1,87) = 8.59, *p* = 0.004, $\eta_{p}^{2}$ = 0.090. Neither ratings of improvised, *F*(1,87) = 2.11, *p* = 0.150, $\eta_{p}^{2}$ = 0.024, nor rehearsed expressions, *F*(1,87) = 1.15, *p* = 0.287, $\eta_{p}^{2}$ = 0.013, were affected by presentation format.

## Discussion

Emotions are a central aspect of social interactions, however, not all expressions of emotion are genuine. Knowing the authenticity of an expression can be a crucial factor in determining our perception of and interaction with others (Johnston et al., 2010). Here, we investigated decoders’ ability to discriminate genuine expressions of surprise from deliberate expressions produced after a recent experience with actual surprise or in its absence, presented both in dynamic and static format. Our results support our predictions, finding significant effects due to both presentation format and expression type. We extend past emotion perception research by considering how different methods of producing an expression can affect perception and authenticity discrimination.

Genuine expressions, when presented dynamically, were perceived both genuine-looking and intense, echoing past findings (Sato and Yoshikawa, 2004; Krumhuber et al., 2013). These were also the most accurately discriminated as having occurred in the presence of an affective event (i.e., seeing the jack-in-the-box) and the most confidently judged by decoders, compared to the two deliberate expression types. In static presentation, genuine expressions were still the most accurately discriminated, but markedly lower than when presented dynamically. Conversely to the alternative presentation, in static format, these were rated as more genuine than rehearsed expressions, but equal to improvised expressions on genuineness. Decoders’ judgmental confidence did not differ between expression types, and was significantly lower than in dynamic presentation.

For the deliberate conditions, in line with our predictions, rehearsed expressions presented dynamically were rated as appearing more genuine than improvised expressions, but still lower than genuine expressions. They were also perceived as less intense than genuine expression, but equal to improvised expressions. Decoders were poor at detecting rehearsed expressions as being deliberate, showing the lowest overall accuracy. Confidence was equal to that of improvised expressions, but still lower than genuine. When presented statically, however, rehearsed expressions were rated lower than improvised expressions in terms of genuineness, but equally on intensity and judgment confidence to genuine expression. Lastly, improvised expressions, in dynamic format, were rated the least genuine-looking of all expressions (rated negatively), but rated equally intense and confidently to rehearsed expressions. These expressions were also poorly discriminated as being deliberate. When presented statically, their intensity ratings were significantly higher than those of all other expressions, confirming our predictions; they also were perceived equally genuine-looking and judged as confidently as genuine expressions.

These findings have important methodological implications for the emotion field. To understand human emotion perception, we argue, considerations must be given to (1) the ability to separate genuine from deliberate expressions of emotions, and (2) differences in how the emotion stimuli are produced, as it is clear that these can significantly impact decoder perception. Presentation format was also an important factor in emotion perception (Hess and Kleck, 1994; Ambadar et al., 2005). Expressions presented dynamically were more accurately discriminated, were judged more confidently, and differences in their perceived intensity and genuineness were more pronounced; static presentation limited such perceptual differences between expressions.

Past inconsistencies reported for decoders’ ability to discriminate expression authenticity (e.g., McLellan et al., 2010; Porter et al., 2012), we suggest, may be resolved by considering the type of expressions used and the presentation format. Here, decoders displayed some perceptual ability in recognizing genuine surprise (static and dynamic), but accuracy was not perfect. While for the deliberate expressions, their ability to discriminate these as not being genuine was poor, in both formats, and varied by expression type (marginally); these performances were even poorer when presenting the expressions as static faces. Decoders, also, showed no self-awareness relating to their accuracy; while they perceived differences in expression intensity, genuineness, and even judgment confidence (predominantly in dynamic presentation), these did not aid authenticity discrimination. Given these performances, it would suggest that decoders do not possess a finely tuned perceptual mechanism to discriminate facial expression authenticity, as they do for emotion categorization.

In the current study, decoders evaluated the expression in the absence of external or contextual information. Eliciting the expressions in a controlled environment permitted a clear comparison between different expression types. However, decoders are unlikely to see such isolated expressions in everyday scenarios with the sole task of detecting authenticity (Reisenzein et al., 2006). This may partly explain why using emotional cues as markers for deception does not produce improvements in accuracy (Porter et al., 2012). Relying on such “cues” will not be beneficial unless decoders can discriminate if these are genuine or deceptive (see Zloteanu, 2015). An interpretation of the current findings is that senders are capable of producing expressions that look sufficiently genuine to fool decoders (Krumhuber and Manstead, 2009; Gunnery et al., 2013). Emotional expressions, thus, can be a strategic tool in communication, used to instill a specific affective belief in the decoder, which benefits the sender. It is not difficult to extend this logic to other deceptive scenarios, such as high-stakes criminal lies, where producing a deceptive expression might help escape suspicion (e.g., Porter et al., 2012). Our findings cast doubt that in a real-world setting where people are not instructed to classify the authenticity of emotional displays, and where emotions tend to more ambiguous, observers could accurately distinguish genuine from deceptive emotional signals. Alternatively, context can, in certain scenarios, aid authenticity judgments (Blair et al., 2010). Removing context from the judgment task may in turn have affected decoders, as the information which may hint that an expression is genuine/fake was absent.

The current consideration for expression type can also aid our understanding of emotion recognition. Intensity is considered an important component in the perception and accurate classification of emotions (Hess et al., 1997). It has been argued that deliberate expressions may appear either *less* intense in presentation, as they are absent of the underlying affect (Levenson, 2014), or *more* intense, as they are attempts by the sender to communicate information successfully (Calder et al., 2000). Given the current results, this may be resolved by considering how the expressions are produced. Namely, rehearsed expression were perceived as less intense than genuine expressions (in dynamic format), while improvised expressions were perceived as more intense (in static format). For this reason, differences on emotion perception tasks may occur based on the authenticity of the stimuli (i.e., genuine or deliberate), the type of production method used (e.g., rehearsed or improvised), the presentation used (i.e., dynamic or static), or a combination of these factors. For instance, using static improvised expressions in a recognition task, due to their perceived high intensity, may result in overinflated recognition rates for surprise. Regarding authenticity discrimination, intensity did not show any relationship with accuracy, in either dynamic or static presentation. Thus, facial intensity seems not to be diagnostic of authenticity, but more related to the method of production used to elicit the expression.

Finally, dynamic presentation of facial displays offers clear benefits to emotion research. Given the current data, it is clear that using ecologically valid stimuli that reflect genuine expressions allow for subtle differences between expression types to be perceived by decoders, and offer a more realistic approximation of human emotion perception (Trautmann et al., 2009; Sauter and Fischer, 2017). Future research should expand the current findings to explore how decoders perceive other emotions, given the variation in perception and accuracy based on valence and category (see Barrett, 1998), and extended to more social emotions, such as shame and embarrassment (e.g., Tracy and Matsumoto, 2008), to better understand emotion production and perception. Expansions may also consider individual differences in expressive control (Berenbaum and Rotter, 1992) and emotion regulation (Gross, 2002) as factors for the successful production of deliberate expressions. Such work may examine how expressive variability relates to perceptual accuracy, by considering an inter-item analysis of the current stimuli or by directly measuring expressive behavior in the task (e.g., using automated facial expression analysis; Valstar et al., 2006). Also, different emotions could have different effects in terms of senders’ ability to display genuine-looking expressions and decoders’ ability to discriminate authenticity. For instance, the current approach did not consider the role of the gender of the sender, which some research suggests may affect perception (e.g., Krumhuber et al., 2006); future research should test for gender differences in production and perception.

## Conclusion

The ability to accurately discriminate and perceive differences in expressions of surprise was affected by both the type of deliberate expressions seen and the way they were presented. Even when asked to specifically judge authenticity, decoders were not adept at separating genuine from deliberate expressions of surprise. While they showed some ability to accurately detect genuine surprise, they also tended to misclassify deliberate expressions as genuine, regardless of expression type. The way in which the deliberate expressions were produced also affected how they were perceived. Rehearsed expressions, in a dynamic format, were perceived as more genuine in appearance than their improvised counterparts and were slightly more difficult to detect as non-genuine. In comparison, improvised expressions were rated as more intense and genuine in appearance in a static format. This supports our predictions of perceptual differences between genuine and deliberate expressions occurring as a result of the method used to produce and present the stimuli. For measuring differences in human emotion perception and accurate authenticity discrimination a dynamic presentation was found to be superior, allowing for nuanced perceptions of intensity, genuineness, and judgment confidence between expressions. Together, the findings illustrate the complexity of human emotion production and perception, the need for ecologically valid stimuli, and the importance of considering expression type in emotion research.

## Author Contributions

MZ and DR conceived and designed the experiments. MZ performed the experiments. MZ and EK analyzed the data. MZ, EK, and DR contributed to writing the paper.

## Conflict of Interest Statement

The authors declare that the research was conducted in the absence of any commercial or financial relationships that could be construed as a potential conflict of interest.

## Footnotes

1 www.mturk.com
